# Frequencies of persistence, activity pacing, fear avoidance and general stress in acute neck pain

**DOI:** 10.1016/j.cpnec.2025.100308

**Published:** 2025-06-18

**Authors:** Morf Rita, Reicherzer Leah, Degenfellner Jürgen, Hasenbring Monika, Erat Anna, Hotz-Boendermaker Sabina

**Affiliations:** a Zurich University of Applied Sciences (ZHAW), School of Health Sciences, Institute of Physiotherapy, Katharina-Sulzer-Platz 9, CH-8400, Winterthur, Switzerland; b Ruhr-Universität Bochum (RUB), Faculty of Medicine, Universitätsstrasse 150, DE-44801, Bochum, Germany; c Hintsa Performance, Lapinlahdenkatu 1 C, Helsinki, Finland; d Pain in Motion Research Group, Belgium^1^

**Keywords:** Neck pain, Activity patterns, Subjective stress, Objective stress, Hair cortisol concentration

## Abstract

**Background:**

Neck pain (NP) is a common musculoskeletal health problem, persisting in 47 % of cases. A person's engagement in daily activities is defined as activity patterns (eustress persistence, distress persistence, activity pacing, and fear avoidance) that influence the development and continuation of pain. These behaviors may be linked to heightened stress levels, further negatively impacting pain perception. Understanding these relationships is vital, given the intricate interplay between stress, pain, and activity patterns. This study aims to assess the frequency of activity patterns and identify their impact on stress levels in participants with acute NP.

**Methods:**

125 individuals aged 18–65 with acute NP were recruited. Activity patterns were identified using the Avoidance-Endurance Fast Screen, which calculated the frequencies of activity patterns. Furthermore, subjective stress was evaluated using the Stress and Coping Inventory and objective stress using hair cortisol concentration to determine which activity pattern group experienced the highest stress.

**Results:**

Most participants were classified in the eustress persistence group (52 %), followed by activity pacing (22.8 %), distress persistence (19.5 %), and fear avoidance (5.7 %). Subjective and objective stress levels consistently remained below established reference values. Analysis of activity pattern groups showed that distress persistence had the highest subjective stress levels, followed by fear avoidance, while activity pacing had the lowest. No relevant differences between the activity pattern groups were observed in the objective stress measurements.

**Discussion:**

To the best of our knowledge, this study represents the first investigation of frequencies of activity patterns and subjective and objective stress in acute NP.

## Highlights

• Most participants were classified in the eustress persistence group (52 %).
• Distress persistence showed the highest subjective stress levels among activity patterns.
• Objective stress levels, measured via hair cortisol, showed no relevant group differences.
• Participants showing activity pacing had the lowest subjective stress levels.
• This study is the first to assess activity patterns and stress levels in acute neck pain.

## 1 Background

With a lifetime prevalence of up to 71 % in the general population worldwide, neck pain (NP) affects a large proportion of the global population [1]. The Global Burden of Disease Study 2021 shows neck pain prevalence rising from 115 million in 1990 to 203 million in 2020, with increased disability, especially in older adults and women [2]. Research on the course of NP has predominantly focused on primary care settings, revealing that 47 % of participants with NP experience ongoing pain after one year [3]. While many individuals experience spontaneous remission shortly after the onset of neck pain, a subset of patients may experience symptom recurrence or worsening over time, indicating a more chronic course of pain for these individuals [4]. Identifying and addressing mediating factors in an acute pain episode may help alleviate persistent pain in clinical practice. Investigating a person's engagement in daily activities, such as activity patterns (eustress persistence, distress persistence, activity pacing, and fear avoidance), is essential for understanding the development and maintenance of persistent pain [5]. Fear-avoidance behavior involves pain catastrophizing and decreased engagement in life activities [6]. Contrarily, persistence entails task continuation despite the pain to the point of severe pain aggravation [7]. The Avoidance Endurance Model suggests a specific subgrouping in persistence behavior: persons with eustress persistence respond with focused cognitive distraction and positive mood, which can initially provide short-term pain relief [7]. In distress persistence, people often exhibit thought suppression, distress, and perseverative behavior [8]. Activity pacing, the third pattern, balances activity and regeneration for favorable outcomes [9]. Extensive research has been conducted in behavioral studies on fear avoidance, highlighting the central role of fear of pain and avoidance of pain-associated activities in the development of chronic pain and related disabilities in everyday life [6]. However, far less attention has been given to persistence and activity pacing. Despite this research gap, emerging evidence suggests that these overlooked patterns play a crucial role in cognitive pain processing and are thought to reflect different self-regulation strategies in pain, such as balancing activity and recovery or continuing behavior despite discomfort [7,9]. Nevertheless, the distribution of activity patterns in the acute pain domain (less than 4 weeks) remains under investigated, particularly among participants with NP. Existing data from participants with subacute low back pain (LBP) indicate that only 10 % exhibit fear avoidance, 36 % persistence (19 % distress persistence, 17 % eustress persistence), and 54 % activity pacing [10]. In high-performance athletes with LBP in the last three months, 9.8 % were found to engage in fear avoidance, a bigger group of 67.1 % (20.1 % distress persistence, 47.0 % eustress persistence) showed persistence, and 23.1 % showed activity pacing [11]. Individuals' daily activities may be impacted by pain, leading to stress and reduced mental capacity. The relationship between individual activity patterns and stress remains an intriguing area of research.

Stress is a physical and psychological response to restore homeostasis in an organism triggered by challenging circumstances [12]. In addition to the rapid sympathetic fight or flight response, the neuroendocrine hypothalamic-pituitary-adrenal (HPA) stress axis serves as a slower defense against stress, leading to the release of cortisol [13]. Cortisol levels are considered objective stress indicators, while questionnaires measure subjective stress [14]. In the context of stress and activity patterns, research in healthy adults on salivary cortisol levels as a response to an experimental pain induction (cold pressor test) indicated a positive correlation between cortisol and fear avoidance and a negative correlation between cortisol and persistence, suggesting that persistence could be advantageous in managing pain [15]. Testing basal adrenocortical activity during the awakening situation, also representing momentary stress, showed that persistence was related to lower cortisol levels in participants with acute LBP; however, emotional distress may enhance cortisol levels [16]. Long-lasting cortisol levels in human hair can be analyzed, which allows a retrospective insight into the last six months [17] and offers the advantage of simple, noninvasive sample collection and stable storage, as hair does not degrade like other body fluids or tissues [18]. Individuals experiencing chronic pain [19,20] and endometriosis [21] exhibit significantly elevated hair cortisol levels in comparison to healthy adults. However, the impact on cortisol levels in acute NP (lasting less than four weeks) remains unclear.

This study aimed to assess the frequencies of activity patterns (eustress persistence, distress persistence, activity pacing, and fear avoidance) and subjective and objective stress levels in participants with acute NP. The second step of the study focused on identifying the activity pattern group with the highest subjective and objective stress levels.

## 2 Methods/Design

### 2.1 Study design

This sub-study is part of a larger cohort study investigating clinical, somatic, and psychosocial factors of NP over one year. For this publication, study appointment 1 was analyzed within four weeks of pain onset. This investigation follows the STROBE guidelines [22] and is in accordance with the Declaration of Helsinki. Approval was obtained from the local ethics committee (BASEC-No. 2022–00846). More information about the study procedures can be found in the published study protocol [23].

### 2.2 Participants

The cohort included 125 participants with acute NP (pain onset less than four weeks), pain-free for the previous three months, aged 18–65 years, and proficient in German. Exclusion criteria were recent pregnancy or childbirth, peripheral or central neurological, oncological, chronic pulmonary, or acute psychiatric conditions. Frequent headaches (≥2x monthly) and migraines (≥1x monthly) were excluded due to their high association with NP and moderate evidence of cervical musculoskeletal impairments [24,25].

### 2.3 Recruitment

Participants were recruited from local physiotherapy practices, healthcare centers, universities, and companies. Interested individuals registered online and underwent an eligibility screening via survey and telephone interview. Of the 639 individuals who registered online, 514 were excluded prior to study enrollment due to non-responses, not meeting the inclusion criteria (as determined during telephone screening), or withdrawal. No data were collected from these individuals. The study flow chart is available in Appendix A. Before the study appointment 1, detailed study information was provided, and informed consent was obtained.

### 2.4 Data collection

Study data were collected and managed using REDCap tools hosted at the Zurich University of Applied Sciences [26,27]. Participants received a personal link to complete the online survey within two days, and reminders were sent if it was not completed within three days. Participants were invited to a clinical testing center to collect hair samples.

### 2.5 Measurements

#### 2.5.1 Baseline characteristics

The participant's sociodemographic variables, educational level, work status, medication, and medical consultations were recorded. Furthermore, NP-related variables such as the Neck Disability Index, which assesses daily life disability [28], and the PainDETECT to measure pain intensity [29] were used. Moreover, psychological variables were measured to provide a broad overview of the participants' initial conditions, capturing a range of factors that could influence their experiences and behaviors during the study. For this purpose, we employed the Depression Anxiety Stress Scale - 21 [30], the Pain Vigilance and Awareness Questionnaire [31], and the State Anxiety Scale from the State-Trait Anxiety Inventory [32] primarily for discriminative purposes. Lifestyle factors, including smoking status, sleep quality, and physical activity levels were also evaluated using the Physical Activity Questionnaire - Short Form [33].

#### 2.5.2 Activity patterns

The Avoidance Endurance Fast Screening tool was selected to assess activity patterns because of its concise and time-efficient format, making it well-suited for clinical use [34]. It was developed from a previous 37-item screening tool (Avoidance-Endurance Questionnaire) [35] by reducing the number of items and scales while maintaining internal validity [34]. The questionnaire consists of nine items, with seven forming the Pain Persistence Scale (PPS) and two constituting the Depressive Mood Scale (DMS). The PPS scale demonstrated a sensitivity of 0.93 and a specificity of 0.81, with an AUC of 0.87 (95 % CI: 0.80–0.93). The DMS scale showed a sensitivity of 0.82 and a specificity of 0.92, with an AUC of 0.87 (95 % CI: 0.80–0.94) [34].

Each item in the PPS is rated on a Likert scale from 0 (never) to 6 (always) and is filled in to capture behaviors for mild and severe pain. The two items in the DMS assess an individual's general mood over the past two weeks. The first item evaluates whether they could still enjoy things as before, with response options: “I can enjoy things just as much as I used to” (scored 0) or “I can no longer enjoy things as much as I used to” (scored 1). The second item examines decision-making difficulty, offering the responses: “I am as decisive as ever” (scored 0) or “I find it harder than usual to make decisions” (scored 1). Based on the established classification criteria [34], the screening questionnaire categorizes participants into one of four activity patterns: eustress persistence, distress persistence, activity pacing, or fear avoidance.

For the PPS, only answers indicating “severe pain” were considered. The mean of all seven items resulted in a score ranging from zero to six. The DMS consists of two items, with a possible sum score ranging from zero to two. Based on the combination of PPS and DMS scores, participants were assigned to one of four mutually exclusive subgroups following the established classification rules [34]:

• Fear Avoidance: PPS <3 and DMS = 2
• Distress Persistence: PPS ≥3 and DMS = 2
• Eustress Persistence: PPS ≥3 and DMS <2
• Activity Pacing: PPS <3 and DMS <2

Both fear avoidance and activity pacing are linked to lower PPS scores; however, they differ in DMS scores – fear avoidance is marked by high distress, whereas activity pacing is correlated with lower distress [36].

#### 2.5.3 Stress

**Subjective measurement:** The Stress and Coping Inventory measures stress subjectively [37]. This questionnaire evaluates an individual's stress state, including current and long-term stress, as well as physical and mental stress symptoms. It contains three scales with nine items with a total of 54 items. This study used three parts: “Stress due to uncertainty”, “Stress due to excessive demands”, and “Physical and psychological stress symptoms”.

The “Stress due to uncertainty” scale consists of 7 items that assess the extent to which participants felt burdened by specific uncertainties over the past three months. Respondents rate their experiences on a Likert scale from 1 (not burdened) to 7 (very heavily burdened). The items address key areas of life, including finances, housing, job/training, partner, health, and personal expectations. The scale demonstrated good internal consistency (α = .72) [37].

The “Stress due to excessive demands” scale comprises 7 items that assess the extent to which participants felt overwhelmed by various events and problems over the past three months. Responses are rated on a Likert scale from 1 (not overwhelmed) to 7 (very overwhelmed). The items address key areas of life, including finances, housing, job/training, partner, health, and personal expectations. The scale demonstrated good internal consistency (α = .69) [37].

The “Physical and psychological stress symptoms” scale comprises 13 items assessing physical and psychological symptoms linked to stress. Participants were asked about symptoms experienced in the past six months, rated on a Likert scale from 1 (strongly disagree) to 4 (strongly agree). The items cover a range of symptoms, including sleep disturbances, stomach pressure, headaches, sadness, lack of motivation, significant weight changes, reduced sexual desire, withdrawal, concentration difficulties, and nightmares. The scale achieves an excellent reliability (α = .86) [37].

**Objective measurement:** Hair Cortisol Concentration measures stress objectively with good test-retest reliability (r = 0.73) [38]. It is robust against hair-related factors such as natural hair color and washing frequency [39]. The hair samples were harvested at the posterior vertex as close to the head as possible and packaged for dispatch according to the specified protocol [17]. In the laboratory, the first 1 cm of hair was analyzed to determine cortisol levels for the most recent month (Cortisol 1 month), while the subsequent 3 cm segment was assessed to reflect cortisol production over the last three months (Cortisol 3 months) [38]. When hair length was insufficient, only the first 1 cm was used. Men generally have higher cortisol concentrations in their hair than women, so it is important to analyze hair cortisol levels separately for men and women [39].

### 2.6 Data analysis

#### 2.6.1 Descriptive statistics

Descriptive statistics were used to present the baseline characteristics of the study sample. Frequencies of activity patterns (eustress persistence, distress persistence, activity pacing, and fear avoidance), subjective and objective stress levels (Stress and Coping Inventory, hair cortisol concentration) were computed. For the subjective measurement of stress, the total score of each of the three Stress and Coping Inventory scales was utilized: “Stress due to uncertainty”, “Stress due to excessive demands”, and “Physical and psychological stress symptoms”. For the objective measurement, hair cortisol concentrations were analyzed for both 1-month and 3-month periods.

#### 2.6.2 ANOVA

Univariate Analyses of Variance (ANOVA) were conducted to explore potential differences among activity patterns for each dependent variable (“Stress due to uncertainty”, “Stress due to excessive demands”, “Physical and psychological stress symptoms” and Hair Cortisol Concentration), followed by posthoc Tukey's HSD tests.

Physical activity was not included as a covariate in the ANOVA due to weak correlations (r = 0.06–0.23) between the IPAQ-SF and the dependent variables. Although physical activity is often considered stress-reducing [40], it can paradoxically function both as an acute stressor and, over time, as a modulator of neuroendocrine stress responses, complicating its interpretation in this context. However, as a sensitivity analysis, ANCOVAs were additionally conducted, including IPAQ-SF as a covariate. The results remained consistent with the original ANOVAs, indicating that physical activity did not substantially alter the group differences observed.

Analyses were conducted using the R statistical software R version 4.2.1; the complete R code is available on GitRepository (https://github.com/RitaMorf/LongNeck-Acute-data). Three participants' data were missing in the analysis of activity patterns and Stress and Coping Inventory. Hair samples were unavailable for nine participants for Cortisol at 1 month: Six from Eustress Persistence, one from Distress Persistence, and two from Activity Pacing. For Cortisol levels after three months; 12 samples were unavailable: Seven from Eustress Persistence, one was from Distress Persistence, and four were from Activity Pacing. To ensure the accuracy of the results, one participant with a cortisol value exceeding 400 pg/mg (an outlier in the eustress persistence group) was excluded from the analysis.

## 3 Results

### 3.1 Sample characteristics

Between January 2023 and May 2024, 125 participants with acute NP (mean age 29.4 ± 9.2 years, 66 % female) were enrolled. Table 1 outlines the sample characteristics, showing that most participants had a university-level education, painkiller consumption was generally low, the disability levels showed minor symptoms (mean NDI = 22.35 %), and pain intensity was moderate (mean NRS = 4.93). Furthermore, participants were highly physically active, with a mean IPAQ-SF score of 4135.7 MET (Metabolic Equivalent of Task) -min/week. This value is substantially above the 3000 MET-min/week threshold, categorizing the sample as 'highly active’ according to IPAQ-SF guidelines [41].

**Table 1:** Baseline sample characteristics.

Baseline sample characteristics			
	Mean (SD)	Range	95 % CI
**Sociodemographic variables**			
Age (y)	29.4 (9.2)	18–65	(27.73, 30.98)
Female (%)	81 (66)		

**Educational level**			
Apprenticeship (%)	9 (7.3)		
Higher education: university (%)	104 (84.6)		
Other (%)	10 (8.1)		

**Work status**			
Unemployed (%)	3 (2.4)		
Part-time (%)	41 (33.3)		
Full-time (%)	31 (25.2)		
In training (%)	48 (39.0)		

**Medication**			
No Medication (%)	32 (26.0)		
Painkillers (%)	20 (16.3)		
Opioids (%)	0		
Antidepressants (%)	2 (1.6)		
Muscle relaxants (%)	4 (3.3)		
Cannabis (%)	3 (2.4)		

**Medical consultations**			
No consultation of medical professionals (%)	51 (41.5)		
General practitioner (%)	24 (19.5)		
Therapy (physio, chiro, psycho, massage) (%)	41 (33.3)		

**Neck pain-related variables**			
Pain: PainDETECT, 0-10	4.93 (1.61)	1.33–8.33	(4.64, 5.21)
Disability: NDI 0–100 %	22.35 (10.05)	4–50	(20.54, 24.17)

**Psychological variables**			
Depression-Scale: DASS21	7.93 (7.82)	0–40	(6.55, 9.32)
Stress-Scale: DASS21	13.62 (8.63)	0–38	(12.10, 15.15)
Anxiety: STAI-S	43.99 (9.88)	23–70	(42.25, 45.74)
Pain Vigilance: PVAQ	36.54 (9.98)	12–63	(34.78, 38.31)
			
**Lifestyle Factors**			
Physical Activity: IPAQ-SF (min/week)	4135.7 (3594.71)	297–19836	(3495.17, 4776.17)
Sedentary activity: IPAQ-SF (min/week)	491.1 (252.51)	60–2400	(446.44, 535.69)
Smoker (%)	10 (8.1)		
Sleep quality: 0-10	4.40 (2.08)	0–9	(4.04, 4.78)

**Activity Patterns: AE-FS**			
Pain Persistence Scale	3.23 (1.12)	1–6	(3.04, 3.43)
Depressive Mood Scale	0.91 (0.77)	0–2	(0.77, 1.05)

**Subjective Stress: SCI**			
“Stress due to uncertainty”	20.65 (8.16)	7–39	(19.23, 22.07)
“Stress due to excessive demands”	17.22 (6.57)	6–42	(16.08, 18.37)
“Physical and psychological stress symptoms”	12.54 (6.50)	1–27	(11.41, 13.67)

**Objective Stress: Hair Cortisol Concentration**			
Cortisol 1 month male	2.72 (0.94)	1.21–4.70	(1.42, 4.01)
Cortisol 3 months male	2.78 (0.96)	0.72–4.83	(1.44, 4.12)
Cortisol 1 month female	3.30 (2.10)	0.29–10.38	(0.40, 6.21)
Cortisol 3 months female	2.93 (2.04)	0.25–8.99	(0.10, 5.76)

Numbers are means (standard deviations) of participants unless stated otherwise.

95 % CI, 95 % confidence interval; AE-FS, Avoidance-Endurance Fast Screen; DASS21, Depression Anxiety Stress Scale; IPAQ-SF, International Physical Activity Questionnaire Short Form; Mean (SD) mean (standard deviation); NDI, Neck Disability Index; PainDETECT, numeric rating scale (0–10); PVAQ, pain vigilance and awareness questionnaire; SCI, Stress and Coping Inventory; STAI-S, State-Trait Anxiety Inventory-State.

### 3.2 Frequencies of activity patterns

**Fig. 1:**
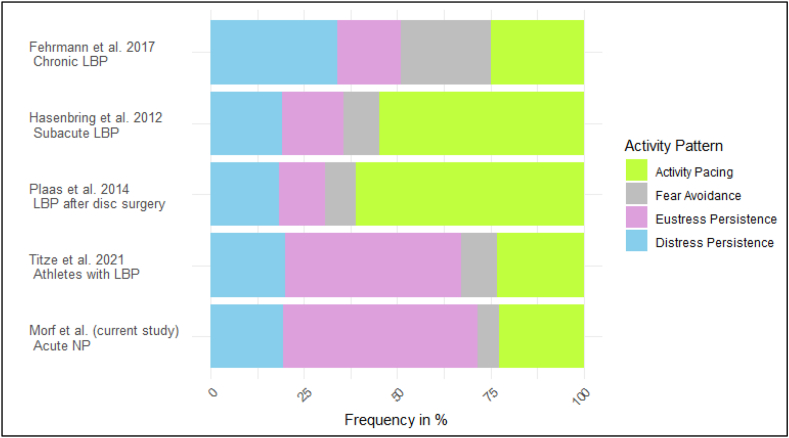
Frequencies of activity patterns in comparison with other study results LBP, low back pain; NP, neck pain.

The distribution of activity patterns showed that the largest group was eustress persistence, with 64 participants (52 %). This was followed by activity pacing with 28 participants (22.8 %), distress persistence with 24 participants (19.5 %), and fear avoidance, which was the smallest with only 7 participants (5.7 %). Fig. 1 presents the distribution of these groups compared to recent findings from other studies.

### 3.3 General stress levels

**Subjective measurement:** All mean scores in our sample were consistently below the established normative values [37]. Mean (SD) scores on the Scale “Stress due to uncertainty” had a mean (SD) total score of 20.65 (8.16), with a 95 % Confidence Interval (CI) of 19.23–22.07. The Scale “Stress due to excessive demands” showed a mean (SD) score of 17.22 (6.57), with a 95 % CI of 16.08–18.37. Especially the items “Performance pressure at work”, “university, training or school” and “Own expectations and demands” revealed the highest mean scores in the whole sample (4.02 and 4.14). The Scale “Physical and psychological stress symptoms” revealed a mean (SD) total stress score of 12.54 (6.50), with a 95 % CI of 11.41–13.67. Additional results for each item can be found in Appendix B.

**Fig. 2:**
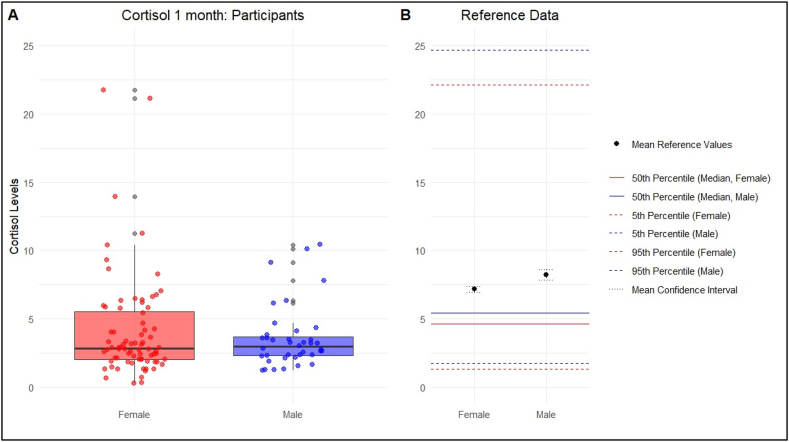
Comparison of objective stress levels (Cortisol 1 month) with the reference values Objective stress levels were measured with the Hair Cortisol Concentration and were separated by gender (female and male).

**Fig. 3:**
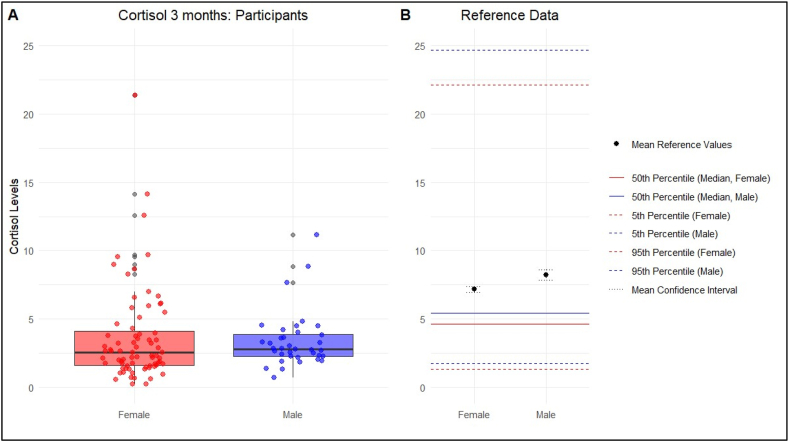
Comparison of objective stress levels (Cortisol 3 months) with the reference values Objective stress levels were measured with the Hair Cortisol Concentration and were separated by gender (female and male).

**Objective measurement:** The cortisol levels observed in the present study were generally lower than the unpublished reference values from a larger cohort of 13′354 participants (mean age 48.1 ± 24.0 years, 69 % female) (Clemens Kirschbaum, personal communication). Fig. 2, Fig. 3 compare the participants' results to the reference values.

**Cortisol 1 month:** The mean cortisol concentration (SE) was 4.14 (0.44) with a 95 % CI of 3.26–5.02 for female participants. The reference data shows a mean (SD) of 7.17 (0.10). The mean (SE) in male participants was 3.60 (0.37), with a 95 % CI of 2.84–4.34.

**Cortisol 3 months:** In female participants, the mean cortisol concentration (SE) over the past three months was 4.13 (0.63), with a 95 % CI ranging from 2.87 to 5.39. The mean (SE) for male participants was 3.40 (0.34), with a 95 % CI of 2.70–4.10.

### 3.4 Stress levels for the individual activity pattern group

**Subjective stress measurement:** The one-way ANOVA examined the effect of activity patterns on the subjective stress level. Overall, distress persistence exhibited the highest subjective stress levels, followed by fear avoidance, eustress persistence, and activity pacing**.** Detailed ANOVA results are presented in Table 2.

**Table 2:** ANOVA results of subjective stress levels.

**ANOVA**				
**“Stress due to uncertainty”**				
	**Mean**	**SD**	**Median**	**P**
Activity Pacing	18.8	7.71	18.6	
Distress Persistence	25.1	7.89	25.4	
Eustress Persistence	19.6	7.77	18.6	
Fear Avoidance	23.9	10.5	24.1	
				0.012
**“Stress due to excessive demands”**				
	**Mean**	**SD**	**Median**	**P**
Activity Pacing	15.3	6.29	14.6	
Distress Persistence	21.3	7.41	20.1	
Eustress Persistence	16.4	5.7	15.9	
Fear Avoidance	17.5	7.49	18	
				0.004
**“Physical and psychological stress symptoms”**				
	**Mean**	**SD**	**Median**	**P**
Activity Pacing	21.8	5.42	20.5	
Distress Persistence	30.2	6.05	32	
Eustress Persistence	25.2	6.05	25.5	
Fear Avoidance	27.9	6.54	29	
				<0.001
**Tukey Post-Hoc Tests**				
**«Stress due to uncertainty”**				
		**95 % Confidence Interval**		
	**Difference**	**Lower Bound**	**Upper Bound**	**P**
Distress Persistence - Activity Pacing	**6.29**	0.54	12.04	0.026
Eustress Persistence - Activity Pacing	0.77	−3.91	5.46	0.973
Fear Avoidance - Activity Pacing	5.08	−3.66	13.82	0.432
Eustress Persistence - Distress Persistence	**−5.52**	−10.47	−0.57	0.022
Fear Avoidance - Distress Persistence	−1.21	−10.09	7.68	0.985
Fear Avoidance - Eustress Persistence	4.31	−3.92	12.54	0.525
**“Stress due to excessive demands”**				
		**95 % Confidence Interval**		
	**Difference**	**Lower Bound**	**Upper Bound**	**P**
Distress Persistence - Activity Pacing	**6.02**	1.45	10.58	0.004
Eustress Persistence - Activity Pacing	1.07	−2.64	4.79	0.875
Fear Avoidance - Activity Pacing	2.20	−4.73	9.13	0.841
Eustress Persistence - Distress Persistence	**−4.94**	−8.87	−1.02	0.007
Fear Avoidance - Distress Persistence	−3.81	−10.86	3.23	0.496
Fear Avoidance - Eustress Persistence	1.13	−5.40	7.66	0.969
**“Physical and psychological stress symptoms”**				
		**95 % Confidence Interval**		
	**Difference**	**Lower Bound**	**Upper Bound**	**P**
Distress Persistence - Activity Pacing	**8.42**	4.11	12.72	<0.001
Eustress Persistence - Activity Pacing	3.44	−0.07	6.94	0.057
Fear Avoidance - Activity Pacing	6.11	−0.43	12.65	0.076
Eustress Persistence - Distress Persistence	**−4.98**	−8.68	−1.28	0.004
Fear Avoidance - Distress Persistence	−2.31	−8.96	4.34	0.802
Fear Avoidance - Eustress Persistence	2.67	−3.49	8.83	0.672

Subjective stress levels were measured with the Stress and Coping Inventory. SD: standard deviation, P: p-value.

Results for the differences between the activity groups are as follows: “Stress due to uncertainty”: F(3, 119) = 3.788, p = .012, eta squared (η^2^) = 0.09. “Stress due to excessive demands”: F (3, 119) = 4.643, p = .004, η^2^ = 0.10 and “Physical and psychological stress symptoms”: F(3, 119) = 9.087, p = <0.001, η^2^ = 0.19.

Post hoc comparisons using Tukey's test showed meaningful differences in subjective stress levels across the activity pattern groups for all three stress scales.

Particularly noteworthy are the scores on the “Stress due to uncertainty” scale: participants with distress persistence scored an average of 6.29 points higher (p = .026) than those with activity pacing. Meanwhile those with eustress persistence scored an average of 5.52 points lower (p = .022) than the distress persistence group.

Notable differences also emerged on the “Stress due to excessive demands” scale: participants with distress persistence scored an average of 6.02 points higher (p = .004) than those with activity pacing. In comparison, those with eustress persistence scored an average of 4.94 points lower (p = .007) than the distress persistence group. Similarly, on the “Physical and psychological stress symptoms” scale, participants with distress persistence reported 8.42 points more (p =<0.001) and 4.98 (p = .004) points more than those with activity pacing and eustress persistence, respectively.

Furthermore, on the 'Physical and psychological stress symptoms' scale, the eustress persistence group scored 3.44 points higher than the activity pacing group (p = .057), and the fear avoidance group scored 6.11 points higher than the activity pacing group (p = .076). These results approached, but did not reach, the conventional significance threshold of p < .05. While not statistically conclusive; these results may indicate meaningful trends, suggesting higher stress symptom scores in both eustress persistence and fear avoidance compared to activity pacing.

### 3.5 Objective stress measurement

A one-way ANOVA examined the effect of different activity patterns on objective stress levels. Results for the differences between the activity groups are as follows: Cortisol 1 month: F(3, 110) = 0.287, p = .835, η^2^ = 0.07, Cortisol 3 months: F (3, 110) = 0.284, p = .837, η^2^ = 0.08. Detailed ANOVA results are presented in Table 3. No Tukey post-hoc tests were calculated because of the non-significant results in the ANOVA.

**Table 3:** ANOVA results of objective stress levels.

**ANOVA**				
**Cortisol 1 month**				
	**Mean**	**SD**	**Median**	**P**
Activity Pacing	3.4	1.82	2.82	
Distress Persistence	4.8	4.78	3.18	
Eustress Persistence	10.3	51.42	2.78	
Fear Avoidance	2.8	0.75	3.2	
				0.834
**ANOVA**				
**Cortisol 3 months**				
	**Mean**	**SD**	**Median**	**P**
Activity Pacing	3.4	1.37	3.25	
Distress Persistence	4.6	3.94	2.65	
Eustress Persistence	11.8	62.90	2.21	
Fear Avoidance	2.6	0.59	2.71	
				0.837

Objective stress levels were measured with Hair Cortisol Concentration. SD: standard deviation, P: p-value.

The calculations of hair cortisol concentration across the activity patterns for cortisol 1 month and cortisol 3 months did not result in relevant differences.

## 4 Discussion

This study first assessed objective and subjective stress and the frequency of activity patterns (eustress persistence, distress persistence, activity pacing, fear avoidance) in persons with acute NP. Then, the differences in subjective and objective stress levels across activity patterns were analyzed.

### 4.1 Frequencies of activity patterns

In our cohort with acute NP, most participants were in the eustress persistence group, followed by activity pacing, distress persistence, and a small number of participants with fear avoidance. The frequencies of activity patterns in our study show preliminary similarities with previous findings in high-performance athletes with and without LBP, suggesting potential parallels in how individuals across varying pain conditions and populations may adjust their behavior in response to pain [11]. However, this is the first study to examine these patterns in individuals with NP. Given the modest sample size, these findings should be interpreted with caution.

Compared to our findings, individuals with subacute LBP [10] or those recovering from disc surgery [42] show a larger activity pacing group and a smaller eustress persistence group. In contrast, studies on chronic LBP report a smaller activity pacing group and a larger amount of fear avoidance and distress persistence group. These findings raise the question of whether fear avoidance behavior in individuals with LBP and NP is genuinely comparable, as LBP is often perceived as more serious than NP in society. Notably, in our sample, fear avoidance behavior is less present than previously assumed, considering prior literature has heavily focused on this activity pattern. In contrast, persistence behavior (distress persistence and eustress persistence) appears to encompass a larger proportion of individuals and should be appropriately recognized and addressed in research and clinical practice. Recent findings in participants with LBP showed that baseline eustress persistence and not avoidance behavior lead to persistent pain [43]. However, it is assumed that an individual can exhibit a combination of fear avoidance and persistence [44]. Activity patterns may fluctuate depending on personal beliefs, motivational factors, and contextual influences [45]. For instance, an individual may engage in physical activities but avoid certain household chores [46].

### 4.2 Stress levels

The low levels of subjective and objective stress observed in our participants may be attributable to several factors. Most participants are highly educated, which is typically associated with higher socioeconomic status. This, in turn, is linked to greater access to material and social resources such as stable employment, healthcare, social support, and flexible working conditions. These factors can reduce exposure to chronic stressors and enhance the capacity to cope with everyday demands. While psychological resilience and adaptive coping may play a role [47], these are likely to be supported and shaped by structural advantages rather than individual traits alone. These contextual factors limit the generalizability of our findings to broader populations.

When examining potential lifestyle influences on cortisol levels, physical activity - generally associated with reduced stress responses [40] - did not show significant correlations in our sample. This may be due to the overall high activity levels and low-stress levels reported by participants. On the other hand, sleep quality was relatively poor in our sample. Poor sleep has been linked to elevated cortisol levels [48,49], which may have contributed to the variability observed in cortisol levels. Furthermore, we did not assess metabolic or nutritional factors. However, studies have shown that elevated hair cortisol is associated with higher BMI, waist circumference, and metabolic changes, suggesting possible links to diet and overall lifestyle [50]. Individual variability in factors such as sleep, physical activity, and unmeasured metabolic influences could contribute to the interpretation of cortisol levels in this sample.

An opposing explanation is hypocortisolism, as reported in individuals experiencing a prolonged period of hyperactivity of the HPA axis due to chronic stress [51]. In such situations, the HPA axis may downregulate cortisol release as the system adapts to prolonged stress, establishing a new baseline. As a result, these individuals may not exhibit typical cortisol responses to stress, requiring more extreme stressors to elicit cortisol release. This adaptation can lead to artificially low cortisol levels, thereby distorting the stress assessment in these participants. This phenomenon has been observed in conditions such as LBP and fibromyalgia [52], severe burnout syndromes [53], atypical depression [54], chronic fatigue syndrome [55]. As none of these diagnoses were excluded in this study, the presence of hypocortisolism in our sample cannot be ruled out. However, the low levels of subjective stress reported suggest that this phenomenon was likely not prevalent among our participants.

### 4.3 Stress and activity patterns

Among the activity patterns, distress persistence exhibited the highest subjective stress levels measured by the Stress and Coping Inventory, followed by fear avoidance, while activity pacing demonstrated the lowest levels overall. These results show that individuals with higher depression scores, such as in distress persistence and fear avoidance, may experience heightened subjective stress levels, at least in terms of “Stress due to uncertainty” and “Physical and psychological stress symptoms”. It is important to note that the second feature of subjective stress, “Stress due to excessive demands”, showed the highest scores in participants with distress persistence, compared to activity pacing and eustress persistence. Especially the items “Performance pressure at work, university, training or school” and “Own expectations and demands” revealed the highest mean scores in the whole sample and, more specifically, in participants with distress persistence, indicating signs of so-called negative perfectionism [56] in this subgroup. Negative perfectionism has been associated with excessive self-criticism, fear of failure, and a focus on mistakes, which may contribute to coping strategies such as catastrophizing and emotional dysregulation [56]. In line with this, research suggests that individuals with difficulties in emotion regulation and low perceived self-efficacy experience higher levels of academic and psychosomatic stress [47]. Interestingly, individuals with distress persistence showed significantly greater difficulties in emotion regulation compared to those with other activity patterns [36]. They also reported higher levels of dysfunctional cognitions such as kinesiophobia and pain catastrophizing, increased affective distress (including depressive symptoms and generalized anxiety) [36] as well as elevated feelings of helplessness and hopelessness [10]. These mechanisms may help explain the elevated stress levels observed in the distress persistence group. In contrast, participants showing more adaptive strategies like activity pacing reported lower stress levels, which may reflect greater self-efficacy and psychological resilience.

The finding that participants with distress persistence show higher stress levels could be relevant in clinical contexts for the early assessment of pain and the planning of interventions to prevent the persistence of acute NP. Although fear avoidance is a well-established prognostic factor in pain chronification, distress persistence may potentially also be associated with poorer outcomes. However, the current study does not allow conclusions about outcomes to be drawn, and further research is needed. In this context, cognitive behavioral therapy and pain education programs may offer helpful frameworks to support individuals in shifting from distress persistence to activity pacing. Although evidence suggests that traditional cognitive behavioral therapy can improve depression, anxiety, and quality of life in people with chronic pain, its effects on pain intensity and pain catastrophizing appear limited [57], highlighting the need further to tailor interventions to behavioral patterns like distress persistence.

Interpreting the calculated differences is also challenging, as no minimal clinically meaningful change has been established for the Stress and Coping Inventory, highlighting the need for further research. Nonetheless, the calculated differences in this study may be considered relevant considering the scale's structure and clinical context. Notably, all differences were found between the same activity patterns across all three subjective stress scales, suggesting that these group distinctions are stable and relevant. Interestingly, differences between the two persistence groups (distress persistence and eustress persistence) were identified, indicating that overacting in daily activities, although with different perceptions, may result in contrary stress levels. These findings could reflect shifts in subjective stress perception related to uncertainty, excessive demands, and stress symptoms. However, the wide confidence intervals observed across group comparisons weaken the robustness of the results. The absence of differences in hair cortisol concentration in our measurements may be explained by the smaller sample size compared to the reference data, potentially resulting in reduced variance.

### 4.4 Strengths and limitations

The measurement and assessment of objective and subjective stress have seldom been conducted in conjunction. This combination of measurements is valuable as it provides insight into a person's subjective perception of their stress level and the body's objective physiological response. The objective stress measurement was limited as not all participants consented to hair sampling, resulting in an incomplete dataset. This limitation weakens the interpretation of the cortisol analyses.

The recruitment through physiotherapy practices proved challenging, as individuals with acute neck pain often do not seek physiotherapy immediately. Consequently, many participants were recruited through mass e-mail invitations at universities and colleges. As a result, critical consideration must be given to the generalizability of the findings, as the sample was predominantly highly educated, physically active, and European, limiting the extent to which the results can be applied to the general population. Therefore, future research should aim to include more diverse samples both geographically and socioeconomically to improve external validity and better reflect the heterogeneity of the global population affected by NP and stress.

The assessment of activity patterns was based on self-reported responses using the AE-FS, which, while psychometrically validated, may not fully reflect observable behavior in daily life. As with other patient-reported outcome measures (PROMs), potential biases such as recall bias, timing effects, and response tendencies may influence how participants interpret and respond to items [58]. Although the AE-FS captures stable behavioral tendencies, its reliance on subjective reporting remains a methodological limitation.

The reference data on cortisol levels is based on a multinational cohort comprising participants from various countries across Europe, North America, and Asia. As this population differs from our sample, direct generalization of the reference values is limited. Furthermore, the reference data were derived from a population with a higher mean age than our sample, which may limit the direct comparability of hair cortisol levels across groups.

Notably, the distinction between significant and non-significant results is based on observed trends and should not be interpreted as a strict dichotomy, in line with recent recommendations for a more nuanced evaluation of findings [59]. In our data, some results approached, but did not cross, the conventional significance threshold of p < .05. While not statistically conclusive, these findings may still reflect meaningful trends, suggesting higher stress symptom scores in both eustress persistence and fear avoidance than activity pacing.

## 5 Conclusion

By highlighting the significance of activity patterns and stress in individuals with acute NP, the results may contribute to advancing treatment approaches and might emphasize the importance of a multidimensional approach to pain management. This study underscores that individuals with persistence behavior is the most prevalent activity pattern in a cohort with acute NP. These findings can provide healthcare professionals with an understanding of patients' behavior towards specific activities and derive treatment interventions to prevent the persistence of acute NP.

## Additional material

As additional material, Appendices 1 and 2, Appendix A are provided.

## CRediT authorship contribution statement

**Morf Rita:** Writing – review & editing, Writing – original draft, Project administration, Methodology, Investigation, Formal analysis, Data curation, Conceptualization. **Reicherzer Leah:** Writing – review & editing, Project administration, Investigation, Formal analysis, Data curation, Conceptualization. **Degenfellner Jürgen:** Writing – review & editing, Methodology, Formal analysis. **Hasenbring Monika:** Writing – review & editing, Methodology. **Erat Anna:** Writing – review & editing, Project administration. **Hotz-Boendermaker Sabina:** Writing – review & editing, Writing – original draft, Project administration, Methodology, Funding acquisition, Data curation, Conceptualization.

## Trial registration

NCT05468684.

## Ethics approval and consent to participate

This study is subject to the Human Research Act (category A, clinical trial with minimal risks). Furthermore, the comprehensive study by the Zurich University of Applied Sciences, part of which this study was reviewed by the Cantonal Ethics Committee on June 21, 2022 and classified as ethically safe and approved (BASEC-No. 2022–00846).

## Consent for publication

Written consent to publish the data was obtained from all participants.

## Availability of data and materials

The data sets used and analyzed in the current study will be available on request from the corresponding author.

## Declaration of generative AI and AI-assisted technologies in the writing process

While preparing this work, the authors utilized DeepL and Grammarly to improve the language quality. Additionally, ChatGPT was employed for assistance with R-coding and language refinement. After using these tools, the authors thoroughly reviewed and edited the content as needed, taking full responsibility for the final version of the publication.

## Funding

This research paper is funded by the 10.13039/501100001711Swiss National Science Foundation [grant number: 32003B_205101/1].

## Declaration of competing interest

The authors declare that they have no conflicts of interest related to this work. There are no financial, personal, or professional relationships that could be perceived as influencing the research, analysis, or conclusions presented in this manuscript.

## List of abbreviations

ANOVA: analysis of variance, CI: Confidence Interval, DMS: Depressive Mood Scale, HPA: neuroendocrine hypothalamic-pituitary-adrenal stress axis, LBP: Low Back Pain**,** MET: Metabolic Equivalent of Task, NP: Neck Pain, PPS: Pain Persistence Scale, PROMs: patient-reported outcome measures (PROMs).
