# Supplementary figures and images for: Frequencies of persistence, activity pacing, fear avoidance and general stress in acute neck pain
Source: Compr Psychoneuroendocrinol. 2025 Jun 18;23:100308. doi: 10.1016/j.cpnec.2025.100308 (PMC12221366; doi:10.1016/j.cpnec.2025.100308)

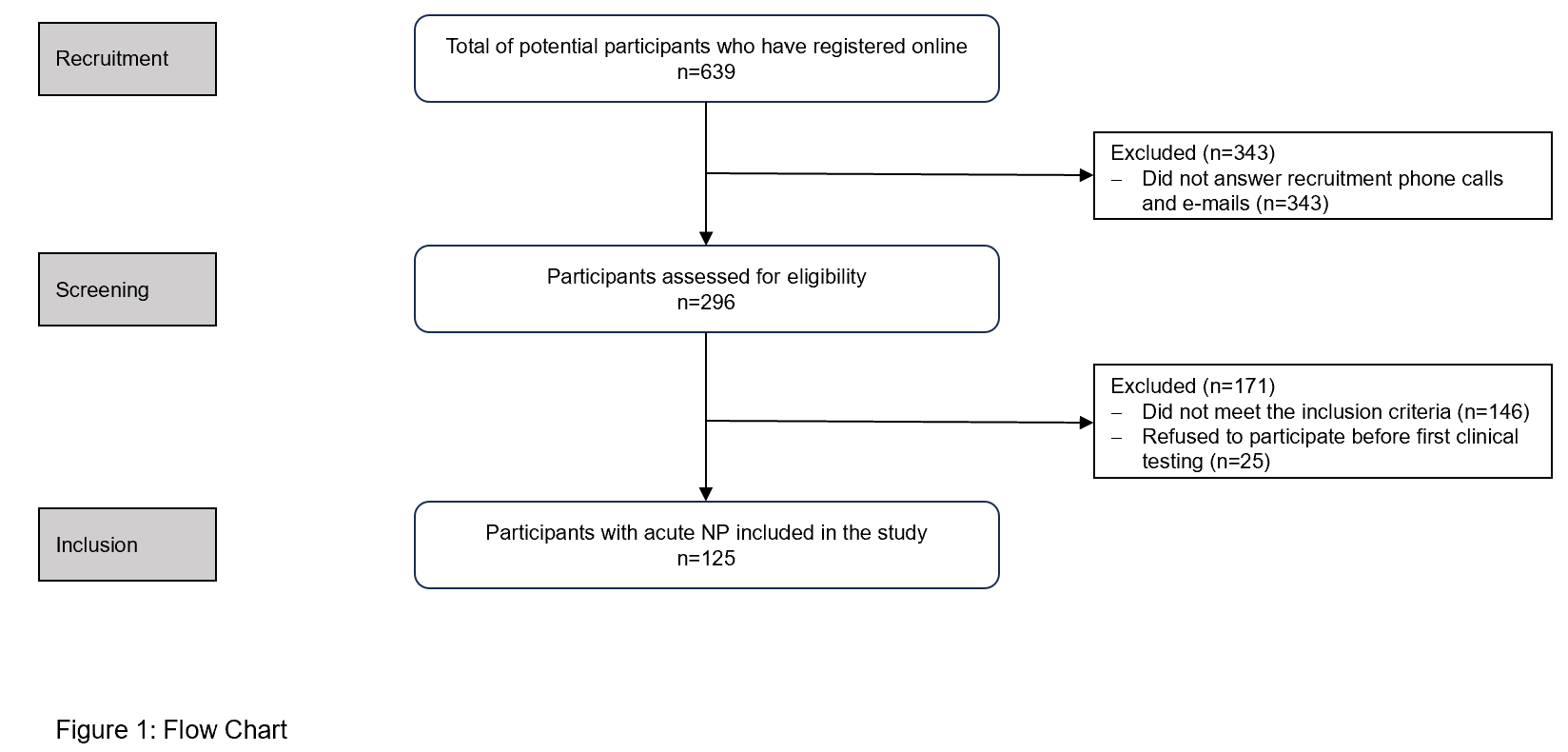
Appendix A: Flow Chart

Supplement: Multimedia component 1 [file mmc1.docx]
