# Supplementary material for: Frequencies of persistence, activity pacing, fear avoidance and general stress in acute neck pain
Source: Compr Psychoneuroendocrinol. 2025 Jun 18;23:100308. doi: 10.1016/j.cpnec.2025.100308 (PMC12221366; doi:10.1016/j.cpnec.2025.100308)
Supplement: Multimedia component 2 [file mmc2.docx]

| **Appendix B:** Stress and Coping Inventory results | | | | |
| --- | --- | --- | --- | --- |
| **Stress and Coping Inventory results** | | | | |
|  | | | | |
| **Scale** | **Mean** | **SD** | **Lower CI** | **Upper CI** |
|  | | | | |
| **Stress due to uncertainty** |  |  |  |  |
| Uncertainty due to financial problems | 3.29 | 1.89 | 2.96 | 3.62 |
| Uncertainty about the place of residence | 2.37 | 1.67 | 2.08 | 2.66 |
| Uncertainty regarding workplace, training place, studies or school | 3.73 | 1.90 | 3.40 | 4.06 |
| Uncertainty in relation to a serious illness | 2.09 | 1.39 | 1.85 | 2.33 |
| Uncertainty in relation to family or friends | 2.92 | 1.82 | 2.60 | 3.23 |
| Uncertainty about partnership | 2.74 | 1.88 | 2.41 | 3.07 |
| Uncertainty regarding important life goals | 3.52 | 1.79 | 3.21 | 3.83 |
| **Total Score** | **20.65** | **8.16** | **19.23** | **22.07** |
|  | | | | |
| **Stress due to excessive demands** | | | | |
| Debts or financial problems | 2.32 | 1.72 | 2.02 | 2.62 |
| Finding an apartment or house building | 1.80 | 1.55 | 1.53 | 2.07 |
| Performance pressure at work, university, training or school | 4.02 | 1.79 | 3.71 | 4.33 |
| Expectations and demands of the partner | 2.28 | 1.64 | 2.00 | 2.57 |
| Health issues | 2.65 | 1.43 | 2.41 | 2.90 |
| Own expectations and demands | 4.14 | 1.75 | 3.83 | 4.44 |
| **Total Score** | **17.22** | **6.57** | **16.08** | **18.37** |
|  | | | | |
| **Physical and psychological stress symptoms** | | | | |
| I sleep badly | 1.63 | 0.89 | 1.48 | 1.78 |
| I often suffer from stomach pressure or abdominal pain | 1.08 | 1.02 | 0.90 | 1.26 |
| I often have the feeling of a lump in my throat | 0.61 | 0.85 | 0.47 | 0.76 |
| I often suffer from headaches | 1.10 | 0.97 | 0.93 | 1.27 |
| I often ponder about my life | 1.56 | 1.05 | 1.38 | 1.74 |
| I am often sad | 0.79 | 0.91 | 0.63 | 0.95 |
| I often lose interest in everything | 0.85 | 0.96 | 0.68 | 1.02 |
| I have lost or gained a lot of weight (more than 5 kg) | 0.57 | 0.84 | 0.43 | 0.72 |
| My desire for sex has decreased significantly | 0.91 | 0.94 | 0.75 | 1.08 |
| I often withdraw into myself and am so absorbed that I don't notice anything anymore | 0.82 | 0.89 | 0.66 | 0.97 |
| I have twitches in my face that I can't control | 0.44 | 0.73 | 0.31 | 0.57 |
| I have trouble concentrating | 1.43 | 0.88 | 1.28 | 1.59 |
| I have nightmares | 0.73 | 0.92 | 0.57 | 0.89 |
| **Total Score** | **12.54** | **6.50** | **11.41** | **13.67** |

Stress and Coping Inventory results

CI, 95% Confidence Interval; SD, standard deviation
